# The open access and dissemination of predatory journals: additional ideas

**DOI:** 10.5935/0004-2749.2024-0177

**Published:** 2024-08-30

**Authors:** Hineptch Daungsupawong, Viroj Wiwanitkit

**Affiliations:** 1 Private Academic and Editorial Consultant, Phonhong, Laos; 2 Department of Pharmaceutical Sciences, University Centre for Research & Development, Chandigarh University Gharuan, Mohali, Punjab, India

Dear Editor:

We would like to provide additional ideas regarding the publication “The open access and dissemination of predatory journals”^(1)^. The proliferation of predatory open access journals and their low publication standards pose a major challenge to the academic community. The business model of predatory journals is based on assisting authors who bear the cost of publication. The medical society may suffer from this practice in several ways, including the publication of unethical reports or the retraction of papers published in lieu of payment. As mentioned, the academic community is concerned about the rise of predatory and low-quality journals in the academic publishing market. These journals frequently accord precedence to financial gain over academic integrity, encouraging the publication of studies that are unethical or unjustly retracting papers that contradict paid publications^(2)^. The publication of poor-quality studies highlights the importance of institutionalizing better control mechanisms for the scientific publishing industry.

In this context, the Scientometrics issue is a striking illustration of the deficiencies in the current retraction policies and editorial integrity^(2)^. The retraction of academic works highlights the need to implement robust and transparent retraction procedures. The instance covered in this article^(2)^ demonstrates how writers are exposed to unwarranted retraction decisions due to the absence of independent oversight and accountability in the scientific publication process. In addition, instances like the author’s qualifications being questioned and conflicts of interest in a published study being brought to light, together with the author’s threats and intimidation on social media, underscore the need for stricter oversight, openness, and regulation in the publishing sector^(3)^. A good example of a problematic case is J Clin Eval Pract, which unethically retracted a letter commenting on the problem of authorship and undisclosed conflict of interest regarding an article authored by non-medical personnel with a vested commercial interest in an anti-COVID-19 vaccine blogpost. This was done to serve the problematic author who publishes in open access mode and has indulged in social network bullying after the retraction of the article.

The aforementioned incidents underscore the necessity of fortifying safeguards for authors and reviewers, enhancing current complaint mechanisms, and guaranteeing impartiality in the management of retractions. The article “The open access and dissemination of predatory journals”^(1)^ underlines the need for a multifaceted strategy to deal with the challenge posed by predatory journals. The scientific community can strive toward developing a more reliable and fair publication system that supports academic freedom and encourages critical thinking by prioritizing integrity, transparency, and responsibility.
